# Prediction of *Microcystis* Blooms Based on TN:TP Ratio and Lake Origin

**DOI:** 10.1100/tsw.2008.89

**Published:** 2008-06-13

**Authors:** Yoshimasa Amano, Motoi Machida, Hideki Tatsumoto, Dennis George, Sharon Berk, Kazuo Taki

**Affiliations:** ^1^ Graduate School of Engineering, Chiba University, Chiba, Japan; ^2^ Center for the Management, Utilization and Protection of Water Resources, Tennessee Technological University, Cookeville, Tennessee, USA; ^3^ Faculty of Engineering, Chiba Institute of Technology, Chiba, Japan

**Keywords:** prediction, TN:TP ratio, lake origin, sediment treatment

## Abstract

We evaluated the relationship between TN:TP ratio and *Microcystis* growth via a database that includes worldwide lakes based on four types of lake origin (dammed, tectonic, coastal, and volcanic lakes). We used microcosm and mesocosm for the nutrient elution tests with lake water and four kinds of sediment (nontreated, MgO sprinkling treated, dissolved air flotation [DAF] treated, and combined treated sediment) in order to control TN:TP ratio and to suppress *Microcystis* growth. *Microcystis* growth was related to TN:TP ratio, with the maximum value at an optimum TN:TP ratio and the minimum values when the TN:TP ratios reached to 0 or ∞. The kurtosis of the distribution curve varied with the type of lake origin; the lowest kurtosis was found in dammed lakes, while the highest was found in volcanic lakes. The lake trophic state could affect the change in the kurtosis, providing much lower kurtosis at eutrophic lakes (dammed lakes) than that at oligotrophic lakes (volcanic lakes). The relationship between TN:TP ratio and *Microcystis* growth could be explained by the nutrient elution tests under controlled TN:TP ratios through the various sediment treatments. A significant suppression of *Microcystis* growth of 70% could be achieved when the TN:TP ratios exceeded 21. Lake origin could be regarded as an index including morphological and geographical factors, and controlling the trophic state in lakes. The origin rather than trophic state for lakes could be considered as an important factor of TN:TP influences on Microcystis growth.

### INTRODUCTION

The outbreak of algae blooms is a worldwide problem[1,2]. Negative effects of algae blooms include odors, fish mortality, and toxin production[3,4,5]. One of the most common nuisance species that forms water blooms is blue-green alga, mainly *Microcystis* species[6,7]. *Microcystis* blooms are related to factors such as light intensity, temperature, and nutrient concentrations, such as nitrogen and phosphorus. These factors have been studied in detail for *Microcystis* growth[8,9,10,11].

The ratio of total nitrogen (TN) to total phosphorus (TP) concentration by weight (TN:TP ratio) influences the occurrence of *Microcystis* blooms. Several studies have reported that *Microcystis* blooms favor the low TN:TP ratio[12,13,14] because *Microcystis* grows well at high phosphorus concentrations[15,16], it is not nitrogen-fixing cyanobacteria, and is thus not a good nitrogen competitor[17,18].

The TN:TP ratio impacts on *Microcystis* imply a balance of nitrogen and phosphorus concentrations, and *Microcystis* could achieve a significant growth at the balanced TN:TP ratio. Contrarily, unbalanced TN:TP ratios could result in the limited growth of *Microcystis*. Neither laboratory nor on-site experiments have shown the quantitative model between TN:TP ratio and the growth of *Microcystis*.

The objective of this study was to reveal the relationship between TN:TP ratio and the growth of *Microcystis* (Chlorophyll a [*Chl.a*] concentration) via a database that includes worldwide lakes based on four types of lake origin (i.e., dammed, tectonic, coastal, and volcanic). A reliability of the relationship was verified through nutrient elution tests by microcosm and mesocosm with lake water and four kinds of sediment: nontreated, MgO sprinkling treated, dissolved air flotation (DAF) treated, and the combination of the MgO and the DAF treatments for the control of TN:TP ratio. The relationship between lake origin and trophic state was also investigated. The importance of lake origin for prediction of *Microcystis* growth is discussed.

### QUANTITATIVE RELATIONSHIP BETWEEN TN:TP RATIO AND *MICROCYSTIS* GROWTH

The growth of *Microcystis* is generally limited by nitrogen concentration at low TN:TP ratios, and also limited by phosphorus concentration at high TN:TP ratios. Alternately, maximum growth is achieved at the balanced TN:TP ratio or the optimum TN:TP ratio[19]. *Microcystis* growth, therefore, would be represented as a type of Gaussian distribution curve as a function of TN:TP ratio, which generally reveals a similar relationship between the gross photosynthetic rate ($P_g$) and light intensity ($I$)[20] as:

$$\frac{P_g}{P_{max}} = \frac{I}{I_{opt}} \exp\left(1 - \frac{I}{I_{opt}}\right) \tag{1}$$

where $P_{max}$ and $I_{opt}$ are the maximum gross photosynthetic rate and the optimum light intensity, respectively. The value of $P_g/P_{max}$ in Eq. (1) increases with increase in $I$ and goes up to the maximum value at $I = I_{opt}$. Afterward, the $P_g/P_{max}$ decreases with increase in $I$ because of the photoinhibition effect.

Under enough solar energy, *Microcystis* growth would be supposed to be represented as follows:

$$\frac{C}{C_{max}} = \left\{\frac{R}{R_{opt}} \exp\left(1 - \frac{R}{R_{opt}}\right)\right\}^{\alpha} \tag{2}$$

where $C$ and $R$ are the *Chl.a* concentration and the TN:TP ratio, $C_{max}$ and $R_{opt}$ are the maximum *Chl.a* concentration and the TN:TP ratio at $C_{max}$, and $\alpha$ is defined as the coefficient expected to differ among the types of lake origins. The physical characteristic of $\alpha$ is described below.

Eq. (2) could be characterized by a grouping of lakes as long as the trophic state is similar, where the maximum *Chl.a* concentration can be used as $C_{max}$ and its TN:TP ratio as $R_{opt}$. In Eq. (2), the distribution curve for $C/C_{max}$ in grouped oligotrophic lakes could entirely show the lower values for the corresponding $R/R_{opt}$, representing that the kurtosis of the distribution would be higher or sharper than that in grouped eutrophic lakes. Hence, the shape of the distribution curve would be expected to differ according to lake trophic state, i.e., TN and TP concentrations. Because the $C/C_{max}$ values for each group of lakes show 1 at $R = R_{opt}$, the kurtosis of the distribution could be represented by $\alpha$. The kurtosis could increase with the increase in the value of $\alpha$. Thus, the variation of trophic state on the grouped lakes could be expressed by $\alpha$ in Eq. (2).

It has been suggested that the lake trophic state would be controlled by the depth, the area of watershed, and the ratio between the area of watershed and surface area[21]. A study for characteristics of water quality on geographical factors reported by Taki and Tanaka[22] indicated that chemical oxygen demand (COD) was in inverse proportion to Secchi disc transparency (SD), and the relationship between COD and SD was featured by lake origin. Based on these reports, the lake trophic state would eventually be controlled by the geographical and physical factor, the so-called lake origin, and accordingly, $\alpha$ in Eq. (2) could be regarded as the coefficient related to the lake origin. Lakes with shallow depth, a large area of watershed, and subsequent large amounts of sedimentary phosphate generally cause a eutrophic condition and, consequently, a high *Chl.a* concentration. For those lakes, $\alpha$ would be given as a low value. On the other hand, oligotrophic lakes and a low *Chl.a* concentration result from a small area of watershed, deep depth, and small amount of sedimentary phosphate. For such lakes, $\alpha$ would show a high value.

*Microcystis* growth, therefore, could be expressed by TN:TP ratio and the coefficient $\alpha$. The finding of $\alpha$ values for lake origins would enable us to predict *Microcystis* growth. In the latter section, the values of $\alpha$ for four types of lake origins are revealed through data analysis of worldwide lakes. The laboratory experiment for the control of TN:TP ratio by means of lake sediment treatment was also conducted to examine the relationship between TN:TP ratio and the growth of *Microcystis*.

### MATERIALS AND METHODS

#### Data Set of the Lakes

The relationship between TN:TP ratio and *Microcystis* growth was examined based on a database of 27 worldwide natural lakes[23,24,25,26,27,28,29,30], which provides algal species including *Microcystis* species, TN and TP concentrations, TN:TP ratio, *Chl.a* concentration, and lake origin. All lakes are listed in Table 1 and include the name, origin, location, trophic state, surface area, mean water depth, water temperature, and pH values. The trophic state for these lakes shows that 22 lakes were eutrophic and five were mesotrophic. The information on origin for these lakes is also represented as 12 lakes that are categorized as dammed lakes, three lakes as tectonic lakes, and six lakes as coastal and volcanic lakes, respectively.

#### Control of TN:TP Ratio by Removing Organic Matter in Sediment

The TN:TP ratio was experimentally controlled by removing organic matter from sediment through the DAF method. The sediment was obtained from Lake Tega, located at 35°50′N, 140°03′E. The lake has a small surface area of 6.5 $km^2$, shallow water depth of 0.9 m, and is well known as one of the most eutrophic lakes in Japan. The sediment was collected using Ekman-Birge–type bottom sampler at the middle part of the lake. The collected sediment was carried to the laboratory and stored at room temperature under the direct rays-free condition.

Fig. 1 shows an apparatus for the DAF treatment. As a procedure of the DAF process, the sediment treatment tank (a) and the circulation tank (b) were filled with 130 l of tap water and microbubbles were generated by the microbubble generator (c). Afterward, 7 kg of the sediment (wet volume) was charged from the top of the sediment treatment tank. At the same time, the inside of the sediment treatment tank was stirred using mixer equipment (d). The beginning of the DAF process was set as 0 min (t = 0) when the sediment was charged, and the flocked and accumulated organic matter (froth) at the upside of sediment treatment tank was removed by aspirating at t = 10, 20, 30, and 40 min. As a coagulant, the polyferric sulfate solution (200 mg-Fe $l^{-1}$) was injected with the dosage of 0.5, 1.0, and 2.5 l at t = 0, 20, and 35 min

**TABLE 1**
**Classifications of Worldwide Natural Lakes where *Microcystis* has Appeared Based on Lake Origin and Trophic State**

| Name | Location | Origin | Trophic state | Surface Area ($km^2$) | Mean water depth (m) | Water temperature (°C) | pH | Reference |
|---|---|---|---|---|---|---|---|---|
| L. Karasu | 39°44'N, 140°05'E | Dammed | mesotrophic | < 1 | 5* | 28 | 8.7 | [23] |
| L. Sohara | 37°41'N, 140°04'E | " | " | < 1 | 5 | 28 | 7.3 | [24] |
| L. Onogawa | 37°40'N, 140°06'E | " | " | 1 | 21* | 25 | 7.3 | " |
| L. Oike | 38°12'N, 138°23'E | " | eutrophic | < 1 | 2 | 26 | 9.2 | " |
| L. Asahi | 37°14'N, 138°22'E | " | " | < 1 | 1 | 33 | 7.8 | " |
| L. Unoike | 37°13'N, 138°21'E | " | " | < 1 | 2* | 32 | 8.5 | " |
| L. Amagaike | 37°13'N, 138°20'E | " | " | < 1 | < 1 | 33 | 9.2 | " |
| L. Hasuike | 36°43'N, 138°30'E | " | " | < 1 | < 1 | 25 | 9.4 | [26] |
| L. Wakuike | 36°35'N, 138°04'E | " | " | < 1 | 7 | 30 | 8.9 | " |
| L. Jonuma | 36°14'N, 139°33'E | " | " | 1 | 1 | 31 | 8.4 | " |
| L. Teganuma | 35°51'N, 140°05'E | " | " | 7 | 1 | 30 | 10.4 | [25] |
| L. Inbanuma | 35°46'N, 140°13'E | " | " | 12 | 2 | 30 | 9.4 | " |
| L. Buhi | 13°05'N, 123°05'E | Tectonic | eutrophic | 17 | 7 | 28 | 8.2 | [29] |
| L. Kinneret | 32°50'N, 35°35'E | " | " | 170 | 26 | 28 | 8.7 | " |
| L. Suwa | 36°03'N, 138°05'E | " | " | 1330 | 5 | 26 | 9.6 | [30] |
| L. Kasumigaura | 36°02'N, 140°24'E | Coastal | eutrophic | 168 | 3 | 27 | 9.3 | [25] |
| L. Kitaura | 36°01'N, 140°34'E | " | " | 34 | 5 | 27 | 8.7 | " |
| L. Sotonakaura | 35°55'N, 140°36'E | " | " | 6 | 9 | 28 | 8.8 | " |
| L. Sanaru | 34°43'N, 137°42'E | " | " | 1 | 1 | 30 | 9.7 | [27] |
| L. Hanareko | 35°41'N, 135°03'E | " | " | < 1 | 3 | 28 | 8.4 | " |
| L. Oike | 32°37'N, 129°50'E | " | " | < 1 | 5 | 29 | 8.7 | [28] |
| L. Rotorua | 38°05'S, 176°15'E | Volcanic | eutrophic | 80 | 11 | 20 | 6.7 | [29] |
| L. Trasimeno | 43°09'N, 12°06'E | " | " | 124 | 5 | 21 | 9.3 | " |
| L. Imutaike | 31°49'N, 130°28'E | " | " | 1 | 1 | 26 | 6.9 | [25] |
| L. Sumiyoshiike | 31°46'N, 130°36'E | " | " | < 1 | 23 | 27 | 8.2 | " |
| L. Ikedako | 31°14'N, 130°34'E | " | mesotrophic | 11 | 126 | 28 | 8.0 | " |
| L. Unagiike | 31°13'N, 130°36'E | " | " | 1 | 35 | 30 | 8.4 | " |

* Maxmum water depth (m)

from the coagulation injection point (e) on the side of the sediment treatment tank. The timing and dosage of the injection provided the highest efficiency to remove the organic matter in the DAF treatment process[31]. After the separation of the organic matter from the sediment, the treated sediment left at the bottom of the sediment treatment tank was collected and used for the nutrient elution tests as described below.

### Control of TN:TP Ratio by Restraining the Nutrient Concentration and by Reducing Organic Matter and Nutrient Concentrations

The restraint of nutrient salts eluted from the sediment was demonstrated through a chemical sprinkling method. The powdered magnesium oxide (MgO) was used as a chemical because MgO would restrain both nitrogen and phosphorus as a form of magnesium ammonium phosphate or magnesium phosphate[32,33], as shown in the following equations:

$$Mg^{2+} + HPO_4^{\ 2-} + 3H_2O \rightarrow MgHPO_4 \cdot 3H_2O \downarrow \quad (3)$$

$$Mg^{2+} + NH_4^{\ +} + HPO_4^{\ 2-} + 6H_2O \rightarrow MgNH_4HPO_4 \cdot 6H_2O \downarrow \quad (4)$$

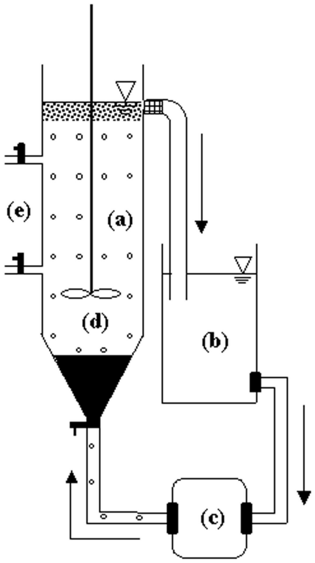

**FIGURE 1.** Set-up for the DAF treatment. (a) Sediment treatment tank, (b) circulation tank, (c) microbubble generator, (d) mixer equipment, (e) coagulation injection point. Arrows represent the direction of water flow.

The reactions shown in Eqs. (3) and (4) proceed under high pH condition. Accordingly, this method would be advantageous for the restraint of nutrient salts in eutrophic lakes because pH value always rises when water blooms occur.

The dosage of MgO on the sediment was 100, 400, and 2000 g $m^{-2}$.

The hybrid method (combining the DAF method and the MgO sprinkling method) was also demonstrated to control TN:TP ratio. The dosage of MgO sprinkled was the same as the MgO sprinkling method.

### Influence of Change in TN:TP Ratio on the Growth of *Microcystis*

#### *Nutrient Elution Test by Microcosm*

There were 100 g of nontreated or DAF-treated sediment separately charged into transparent glass vessels of 470-ml volume. Afterward, lake water containing blue-green algae, mainly *Microcystis* species, obtained at Akebono Bridge, Lake Tega, was added by siphoning, using a rubber tube with a diameter of 1 mm. Since the nondimensional ratio between water depth and sediment thickness in Lake Tega was approximately 4 according to the article reported by Kobayashi and Kusuda[34], the ratio for the nutrient elution test was determined with the same condition for Lake Tega. Each glass vessel was covered using a transparent plastic sheet, and incubated under light ($2 \times 10^4$ lux) and dark (0 lux) conditions with an incubation temperature of 20°C.

The experimental runs were set as shown in Table 2. Run 1 was incubated three times. The results of analyses for each water quality were represented as a mean value for $n = 3$.

#### *Nutrient Elution Test by Mesocosm*

There were 7 kg of nontreated or DAF-treated sediment separately charged into transparent acrylic reactors (70-l volume). Then the raw water was added by siphoning, using a rubber tube with a diameter of 1 cm. The reactors were set outside without any covers.

**TABLE 2**
**Experimental Runs for the Nutrient Elution Test***

| Run | Non-treated | DAF treated | MgO treated | | |
|---|---|---|---|---|---|
| | | | MD: 100 | MD: 400 | MD: 2000 |
| Run1 | ○ | | | | |
| Run2 | ○ | | ○ | | |
| Run3 | ○ | | | ○ | |
| Run4 | ○ | | | | ○ |
| Run5 | | ○ | | | |
| Run6 | | ○ | ○ | | |
| Run7 | | ○ | | ○ | |
| Run8 | | ○ | | | ○ |
| RunI | ○ | | | | |
| RunII | ○ | | | ○ | |
| RunIII | | ○ | | | |
| RunIV | | ○ | | ○ | |

* Runs 1–8: elution test by microcosm; Runs I–IV: elution test by mesocosm. MD, MgO dosage (g $m^{-2}$).

The experimental runs (Runs I–IV) for the nutrient elution test using mesocosm were the same as conditions with Runs 1, 3, 5, and 7 as shown in Table 2. Run I was incubated two times. The results of analyses for each water quality were represented as a mean value for $n = 2$.

### Water Analyses

To examine the effect of the removal of organic matter, the ignition loss (I.L., %) and the grain distribution (μm) were measured by ignition loss method and grain size distribution method, respectively. TN (mg $l^{-1}$), TP (mg $l^{-1}$), and *Chl.a* concentrations (μg $l^{-1}$) were analyzed on the first and last day in the nutrient elution experiment with spectrophotometric method, ascorbic acid method, and spectrophotometric method, respectively.

## RESULTS

### Characteristic of Water Quality Based on Lake Origin

The values of TN, TP, *Chl.a* concentrations, and TN:TP ratio in accordance to lake origin are summarized in Table 3. The mean TN, TP, and *Chl.a* concentrations ($TN_{mean}$, $TP_{mean}$, and $C_{mean}$) were one order of magnitude higher for dammed, tectonic, and coastal lakes than for volcanic lakes. The TN' and TP', defined as the concentrations at $C_{max}$, clearly varied with lake origin, representing that the concentrations were higher in dammed lakes and tectonic lakes, and then decreased in the order of coastal lakes and volcanic lakes, which was proportional to the corresponding $C_{max}$ values. The relationship between *Microcystis* growth and TN:TP ratio is illustrated in Fig. 2. Data plots of *Chl.a* concentration were entirely scattered for

**TABLE 3**
**Characteristics of Water Qualities for the Lakes Used in this Study***

| | Dammed | Tectonic | Coastal | Volcanic |
|---|---|---|---|---|
| $TN_{mean}$ (mg $L^{-1}$) | 1.43 | 0.98 | 1.38 | 0.26 |
| TN' (mg $L^{-1}$) | 2.40 | 2.54 | 1.05 | 0.31 |
| $TP_{mean}$ (mg $L^{-1}$) | 0.103 | 0.124 | 0.137 | 0.027 |
| TP' (mg $L^{-1}$) | 0.333 | 0.360 | 0.100 | 0.023 |
| $R_{mean}$ | 22 | 17 | 16 | 18 |
| $R_{opt}$ | 7 | 7 | 11 | 14 |
| $C_{mean}$ (μg $L^{-1}$) | 73 | 64 | 60 | 7 |
| $C_{max}$ (μg $L^{-1}$) | 316 | 217 | 122 | 23 |

* Sample size: *n* = 21 for dammed lake, *n* = 14 for tectonic lake, *n* = 13 for coastal lake, *n* = 20 for volcanic lake. $TN_{mean}$, $TP_{mean}$, $R_{mean}$, and $C_{mean}$; mean TN, TP, TN:TP ratio, and *Chl.a* concentration, TN' and TP'; TN and TP concentration at $C_{max}$, $R_{opt}$ and $C_{max}$; TN:TP ratio at $C_{max}$ and maximum *Chl.a* concentration.

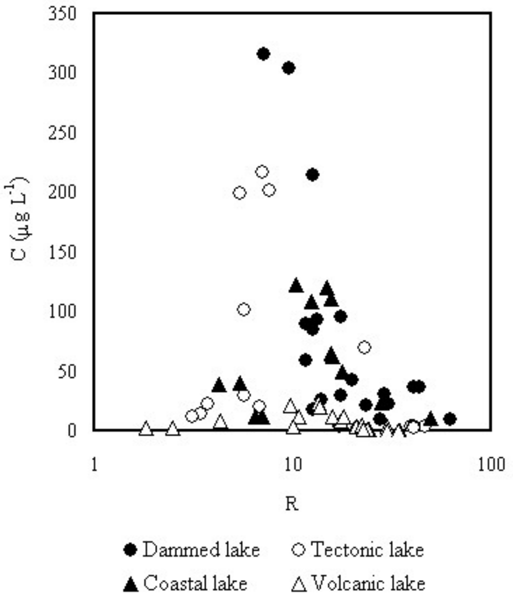

**FIGURE 2.** Relationship between *Chl.a* concentration and TN:TP ratio in the lakes used in this study.

TN:TP ratios. The mean TN:TP ratios ($R_{mean}$) and the TN:TP ratios at $C_{max}$ ($R_{opt}$) showed similar values, but the $R_{opt}$ values for each lake origin were somewhat lower than the $R_{mean}$ values.

### Removal Efficiency of Organic Matter by DAF Treatment

The efficiency of the removal of organic matter from the sediment by the DAF treatment was evaluated by the I.L. and $D_{50}$ as tabulated in Table 4. The I.L. value in the raw sediment for Lake Tega was 15.9% ($n$ = 12), whereas the value for the DAF-treated sediment was 15.1% ($n$ = 12), representing a significant decrease ($p < 0.05$) in the organic matter than that obtained from the raw sediment. The $D_{50}$ in the raw sediment was 6.8 μm, while that in the DAF-treated sediment was 40 μm. Thus, DAF treatment could remove approximately 5% of the organic matter from the raw sediment that would be small particles.

**TABLE 4**
**Influence of the DAF Treatment on Removal of Organic Matter in Sediment**

| Treatment | Ignition loss (%) | Mean diameter (μm) |
|---|---|---|
| Non-treated | 15.9 | 6.8 |
| DAF treated | 15.1 | 40 |

### Influence of Sediment Treatments on the Change in Nutrient Salts and TN:TP Ratio

Change in nutrient salts eluted from the sediment and the corresponding TN:TP ratio through the sediment treatments are illustrated in Fig. 3, based on the microcosm test. The restraint efficiencies of TN and TP could be seen in all of the sediment-treated runs compared with the nontreated run. Here, the restraint efficiency of nutrient salts was calculated as [100–{(concentration for sediment treated run / concentration for nonsediment treated run)} × 100]. In the MgO sprinkling treatment (Runs 2–4), the restraint efficiencies of TN concentrations were in the range between 29 and 73%, except Run 3 (–10%), while significant restraint efficiencies of TP concentrations were found for all runs except Run 6 (17%), and the efficiency for TP concentration increases with the increase in MgO dosage. Consequently, TN:TP ratios for the sediment-treated runs showed higher values (21 < TN:TP ratio < 107), although Runs 5 and 6 remained at low values (TN:TP ratio = 9 for Run 5, TN:TP ratio = 7 for Run 6).

Although less restraint efficiencies of TN concentrations were observed in the nutrient elution test using mesocosm for all treated runs (below 30 %) as displayed in Fig. 4, significant restraint efficiencies of TP concentrations were represented for all runs. Accordingly, TN:TP ratios for all runs were raised and ranged between 20 and 94%, representing the same trends as with the nutrient elution test by microcosm.

### Influence of Change in TN:TP Ratio on the Growth of *Microcystis*

The relationship between $C/C_{max}$ and $R/R_{opt}$ by lake origin, based on Eq. (2) and using the values listed in Table 3, is illustrated in Fig. 5. The plots by lake origin were well fitted to the model expressed in Eq. (2) with the correlation coefficients of 0.88, 0.69, 0.80, and 0.85 for dammed, tectonic, coastal, and volcanic lakes, respectively. The $\alpha$ values can be determined by the best-fitted curve to the resultant values for lake origin. The $\alpha$ values for dammed, tectonic, coastal, and volcanic lakes were calculated to be 8.5, 11.0, 11.5, and 18.5, respectively, which showed negative correlation with the nutrient concentrations for each lake origin. When our data for the nutrient elution tests were applied to the model, the relationship between

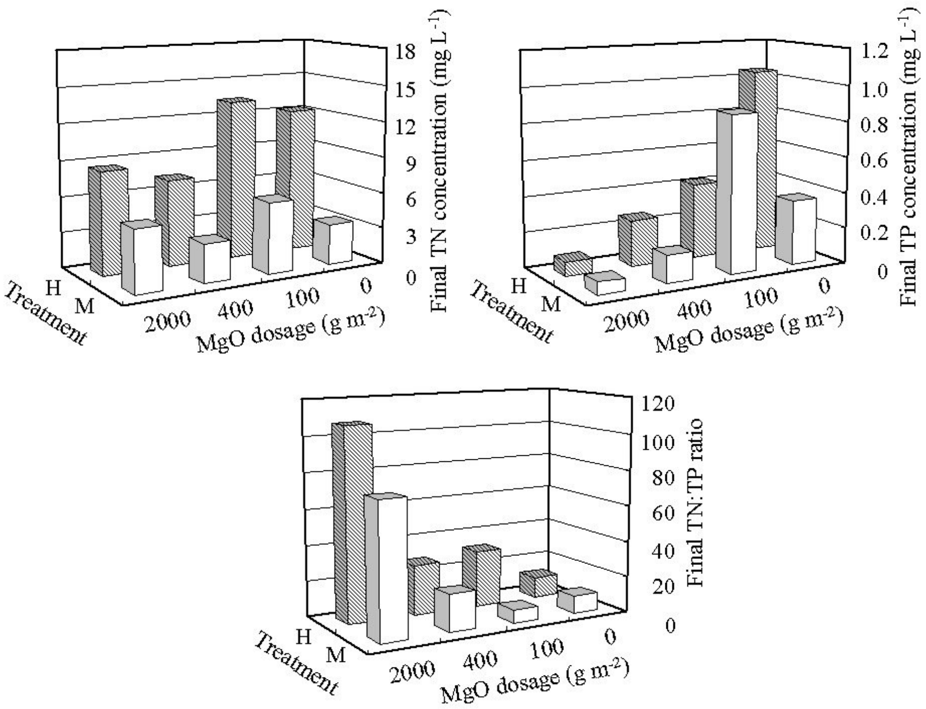

**FIGURE 3.** Changes in the final TN and TP concentrations, and the TN:TP ratios through the nutrient elusion test (microcosm). N, nontreated sediment; M, MgO-treated sediment; D, DAF-treated sediment; H, hybrid-treated sediment.

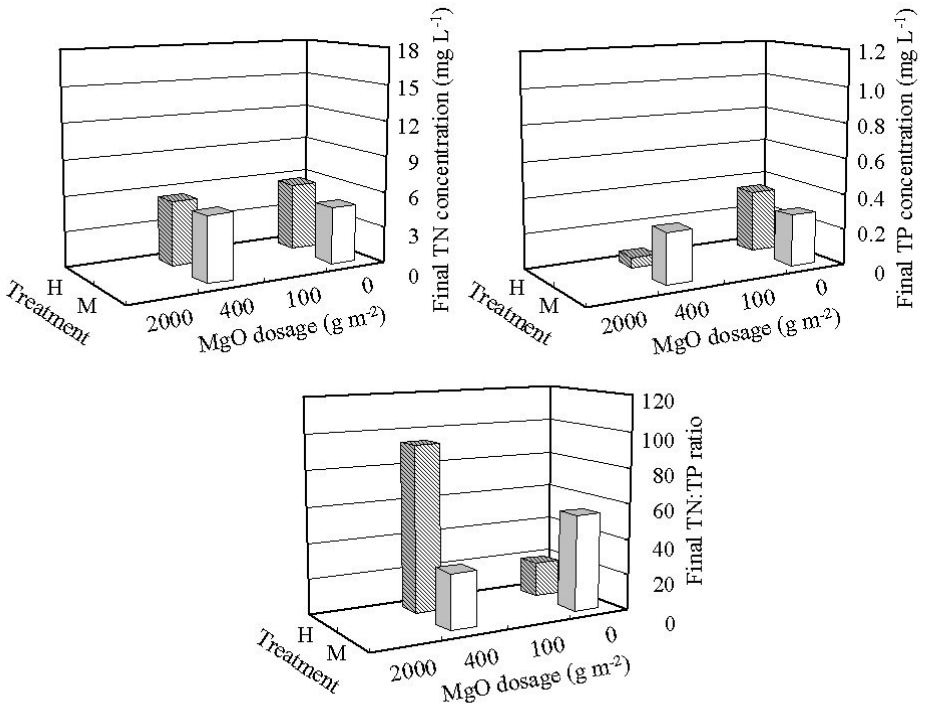

**FIGURE 4.** Changes in the final TN and TP concentrations, and the TN:TP ratios through the nutrient elusion test (mesocosm). N, nontreated sediment; M, MgO-treated sediment; D, DAF-treated sediment; H, hybrid-treated sediment.

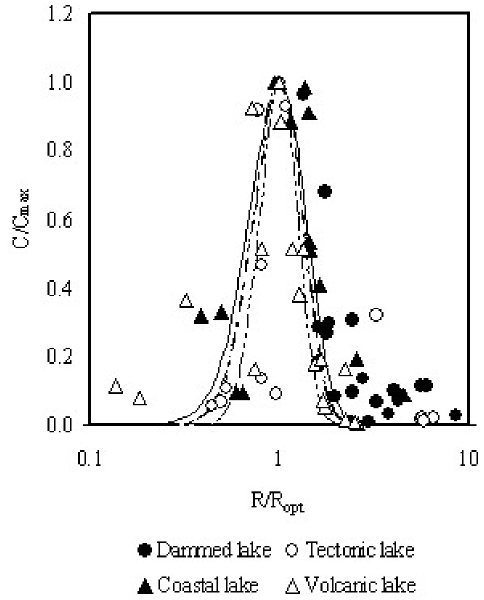

**FIGURE 5.** Data plots of *Chl.a* concentrations and TN:TP ratios by Eq. (2) for each lake origin. Each approximation curve is represented by: solid curve for dammed lake ($\alpha$ = 8.5, $r$ = 0.88), dashed curve for tectonic lake ($\alpha$ = 11.0, $r$ = 0.69), dash-dotted curve for coastal lake ($\alpha$ = 11.5, $r$ = 0.80), and dash-double dotted curve for volcanic lake ($\alpha$ = 18.5, $r$ = 0.85).

TN:TP ratios and *Microcystis* growth could be represented as in Fig. 6, where the $C_{max}$ and $R_{opt}$ for the nutrient elution tests were based on the values of nontreated runs. In the figure, the data for dammed lakes were also added because the sediment and lake water for the nutrient elution test was obtained from Lake Tega, of which origin was specified as a dammed lake.

In Fig. 6, the distribution of $C/C_{max}$ based on the sediment treatments was in correspondence with the model ($r$ = 0.73), representing that *Chl.a* concentrations decreased with increase or decrease in the TN:TP ratios. Also, the data clearly indicated that approximately 90% of *Chl.a* concentrations in Runs 7 and 8 were suppressed compared with the nonsediment-treated run (Run 1).

### DISCUSSION

#### Relationship Between TN:TP Ratio and *Microcystis* Growth

Considerable studies have dealt with blue-green algae and/or *M. aeruginosa* growth based on the TN:TP ratio. Our previous study[13] discovered, through the culture experiment with lake sediment and lake water, that blue-green algae were the dominant algal species when the TN:TP ratios were between 5 and 18 for the summer season. Fujimoto et al.[10] compared the growth abilities for *M. aeruginosa* and *Phormidium tenue* through the semi-continuous culture under various N:P supply ratios, and found that *M. aeruginosa* dominated when N:P ratios were less than 20. Furthermore, in a study of 17 lakes by Smith[12], blue-green algae tended to appear at TN:TP < 29. In contrast to those findings, Xie et al.[14] concluded that *Microcystis* blooms appeared not to be a function of the TN:TP ratios. However, their data showed that the reproduction of *Microcystis* blooms was optimal when the TN:TP ratios were approximately 8 to 9, which agrees with the findings (<10) of Takamura et al.[35].

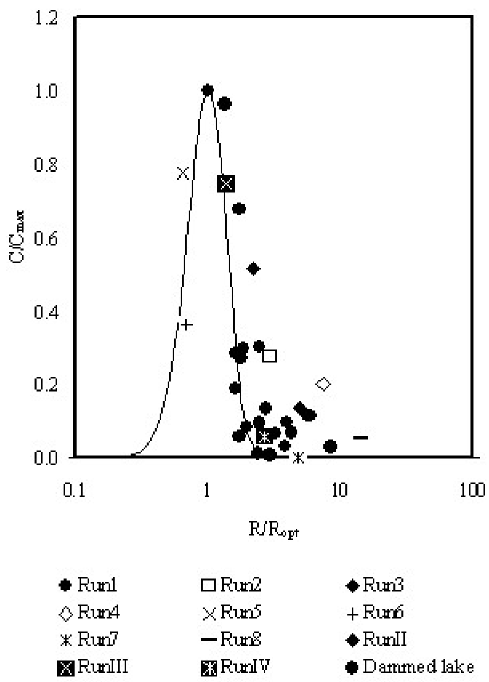

**FIGURE 6.** Influence of changes in TN:TP ratios by each sediment treatment on *Chl.a* concentrations. Solid curve represents the estimated distribution of $C/C_{max}$ for dammed lake ($\alpha$ = 8.5).

In our data analysis for lake origins, approximately 70% of the data used were in the range of 5 < TN:TP < 29. Moreover, the $R_{opt}$ values were in correspondence with the field observations that *Microcystis* blooms experience a significant growth as mentioned above, which agrees with the results obtained from the nutrient elusion tests (Run 1: $R_{opt}$ = 12, $n$ = 3, for microcosm; Run I: $R_{opt}$ = 19, $n$ = 2, for mesocosm). Our results could follow the conventional TN:TP considerations that *Microcystis* could achieve a significant growth where the TN:TP ratio was under low condition, and could support the hypothesis that *Microcystis* growth could be expressed as a function of TN:TP ratio, i.e., *Microcystis* could significantly grow at the optimum TN:TP ratio and decrease with decreasing or increasing the TN:TP ratio.

### Influence of the Factors Related to *Microcystis* Growth

There are possibilities of factors other than the TN:TP ratio that may influence *Microcystis* growth. A trend of increase in algal growth with increasing nutrient concentration has widely been found in many studies[13,16]. This fact indicates that the same TN:TP ratio with different nutrient concentrations considerably causes the variance of *Microcystis* biomass, which is reflected as the relationship between *Chl.a* concentration and TN:TP ratio. The scattering of *Chl.a* concentrations at the same TN:TP ratio could be converged via standardization that the maximum values of $C/C_{max}$ and the corresponding values of $R/R_{opt}$ for each group of lakes by the origin were set to be 1. Then, the influence of nutrient concentrations would be reflected as the kurtosis for $C/C_{max}$ distribution or $\alpha$ value. For example, the $C/C_{max}$ values at the same $R/R_{opt}$ show higher in dammed lakes than in volcanic lakes, implying the difference in trophic state. Thus, the nutrient concentrations would affect the $C/C_{max}$ distribution and/or $\alpha$ value.

Although *Chl.a* data were used to represent *Microcystis* growth, the data included algal species other than *Microcystis*, implying that algal species could influence the relationship between $C/C_{max}$ and $R/R_{opt}$. In the competition experiment carried out by Fujimoto et al.[10] and Holm and Armstrong[16], the biomass of *M. aeruginosa* was one or two orders higher than that of the competitor when the dominant species was *M. aeruginosa*. Similarly, a significant abundance of *Microcystis* biomass was found in a field observation in a eutrophic lake[35]. The *Chl.a* data used in this study included a dominant species of *Microcystis*, so that a high rate of *Microcystis* dominance for the *Chl.a* data used in this study could be expected.

Fujimoto et al.[10] examined the difference of the growth rate of *M. aeruginosa* under different temperatures and found the significant influence of temperature on the growth rate in both phosphorus-limited medium (0.46 day$^{-1}$ at 20°C and 0.88 day$^{-1}$ at 30°C) and nitrogen-limited medium (0.84 day$^{-1}$ at 20°C and 1.45 day$^{-1}$ at 30°C). Jin et al.[36] discussed the influence of pH on the release of phosphorus from sediment. Their experimental results clearly show that pH prompted the phosphorus release, and a remarkable release of phosphorus occurred under pH < 5 and under pH > 7. Thus, *Microcystis* growth, i.e., the distribution of $C/C_{max}$, could remarkably be influenced by both temperature and pH. The mean water temperature and pH for the lakes used in this study were 28°C and 8.6, respectively, which follows the widely accepted facts that *Microcystis* tends to be abundant at high temperature conditions and, thereby, tends to raise the pH value in lake water. The $C/C_{max}$ distribution represented in this study could be regarded as the result of the substantially constant high water temperature and pH, and these conditions would, therefore, cause little influence on the $C/C_{max}$ distribution.

### Lake Origin Controlling Lake Trophic State

Several studies have discussed the factors concerning the trophic state in lakes. Taki and Tanaka[22,37] reported the water qualities for 62 Japanese lakes from the viewpoint of lake origin. A part of their studies can be illustrated in Fig. 7 with some arrangements, i.e., where data other than lakes they used were added. Also, the index represented as the ratio between COD and SD was adopted in this figure to indicate lake trophic state more clearly.

It is obvious that the difference in the COD/SD values could be recognized among lake origins, being much higher values in dammed and coastal lakes than in tectonic and volcanic lakes. This trend would effectively agree with the nutrient concentrations for the lakes used in this study. Fig. 7 also represents the difference in population that followed in order of coastal, dammed, tectonic, and volcanic lakes. Volcanic lakes would significantly be influenced by population in watershed per surface area compared with other types of origin, implying a high sensitivity to even slight increases in the population. Conversely, the COD/SD values in dammed, coastal, and tectonic lakes show the scattered plots against the population. This would indicate the influences of factors other than the population, e.g., surface area, depth, area of watershed, and probably the amount of phosphate in sediment. These factors could be attributed to lake origin, of which consideration would be supported by the reports from Goldman and Horne[21].

The difference of trophic states in lakes would result from an interaction between size and area of watershed for lakes, and these factors would be characterized by lake origin. Lake origin could be regarded as an index including morphological and geographical factors, and controlling the trophic state in lakes, so that the origin rather than the trophic state for lakes could be considered as an important factor of TN:TP influences on *Microcystis* growth.

### CONCLUSIONS

This study reveals the relationship between TN:TP ratio and the growth of *Microcystis* via a database that includes worldwide lakes based on four types of lake origin. The nutrient elution tests using microcosm and mesocosm with lake water and four kinds of sediment were also conducted in order to control TN:TP ratio and to suppress *Microcystis* growth. The main conclusions could be summarized as follows:

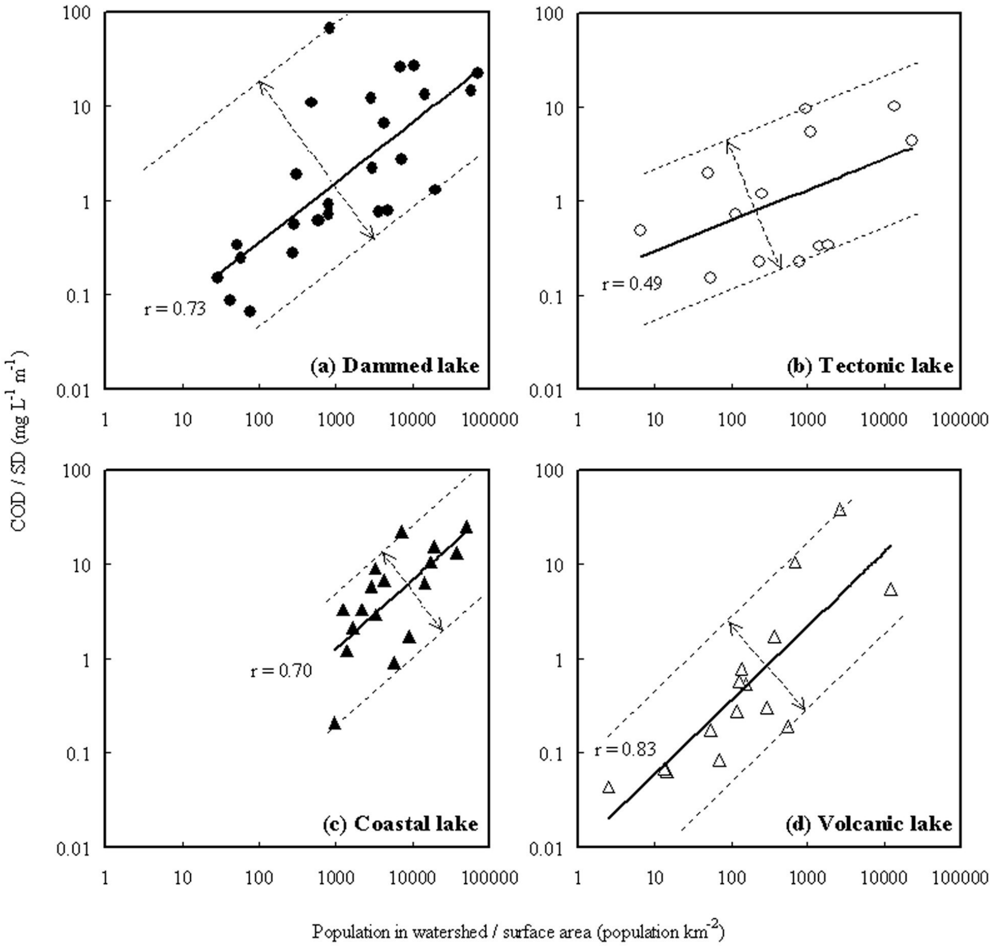

**FIGURE 7.** Difference in trophic state represented from the viewpoint of lake origin. Data plots were cited from the articles reported by Taki and Tanaka[22,37] with some arrangements. Sample size: *n* = 26 for dammed lake, *n* = 13 for tectonic lake, *n* = 17 for coastal lake, *n* = 15 for volcanic lake.

1. *Microcystis* growth could be expressed by TN:TP ratio and described as a distribution curve showing the maximum value at an optimum TN:TP ratio and the minimum values at the TN:TP ratio nearly equal to 0 or $\infty$. The kurtosis of the distribution curve varied with the type of lake origin and could be expressed by the $\alpha$ value.
2. The $\alpha$ values for dammed, tectonic, coastal, and volcanic lakes were calculated to be 8.5, 11.0, 11.5, and 18.5, respectively. The lake trophic state affected the change in $\alpha$ value, providing the much lower $\alpha$ value at the eutrophic state (dammed lakes) than that at the oligotrophic state (volcanic lakes).
3. The nutrient elution tests controlling the TN:TP ratios through the various sediment treatments showed that a significant suppression of *Microcystis* growth (70%) could be achieved when the TN:TP ratios exceeded 21.
4. Lake origin could be regarded as an index including morphological and geographical factors, and controlling the trophic state in lakes. Therefore, the origin rather than the trophic state for lakes could be considered as an important factor of TN:TP influences on *Microcystis* growth.

### ACKNOWLEDGMENTS

We would like to thank Prof. Dr. Masami Aikawa (Kisarazu National College of Technology), Mrs. Yoko Fujimura (Chiba Prefectural Environmental Research Center), and Dr. Tohru Yabe (National Institute for Environmental Studies) for many discussions about the contents of this study. We would also like to express our sincere thanks to the anonymous reviewers for their useful comments and suggestions.
